# Associations of Vitamin B6 Intake and Plasma Pyridoxal 5′-Phosphate with Plasma Polyunsaturated Fatty Acids in US Older Adults: Findings from NHANES 2003–2004

**DOI:** 10.3390/nu14112336

**Published:** 2022-06-02

**Authors:** Hyojung Kim, Evelyn B. Enrione, Vijaya Narayanan, Tan Li, Adriana Campa

**Affiliations:** 1 Department of Dietetics and Nutrition, Robert Stempel College of Public Health and Social Work, Florida International University, Miami, FL 33199, USA; enrionee@fiu.edu (E.B.E.); vnarayan@fiu.edu (V.N.); campaa@fiu.edu (A.C.); 2 Department of Biostatistics, Robert Stempel College of Public Health and Social Work, Florida International University, Miami, FL 33199, USA; tanli@fiu.edu

**Keywords:** older adults, vitamin B6, polyunsaturated fatty acids, pyridoxal 5′-phosphate, vitamin B6 intake, gender, NHANES

## Abstract

Previous evidence suggests a potential dual impact of aging and vitamin B6 (B6) deficiency on polyunsaturated fatty acid (PUFA) metabolism; gender may influence PUFA biosynthesis. Perturbation of PUFA compositions during B6 deficiency could be linked to age-related health outcomes. However, little is known about the interrelationships between vitamin B6, PUFA, and gender in the older population. Therefore, we investigated whether gender-specific associations of B6 intake and plasma pyridoxal 5’-phosphate (PLP) concentration, respectively, with plasma PUFA concentrations and ratios (eicosapentaenoic acid (EPA), docosahexaenoic acid (DHA), arachidonic acid (AA), EPA + DHA, EPA/AA, and (EPA + DHA)/AA) existed in older adults. We further examined the relationships of adequate B6 status (PLP ≥ 20 nmol/L) with high (above median) plasma PUFA relative to deficient B6 status. This cross-sectional study analyzed 461 participants aged ≥60 years from NHANES 2003–2004. Nutrient intakes were assessed using two 24-h recalls and supplement questionnaires. PLP and PUFA concentrations were measured. Multivariate linear regression assessed the association of B6 intake and PLP with PUFA; multivariate logistic regression evaluated the relationship of adequate B6 status with high plasma PUFA, adjusting for demographic, socioeconomic, and dietary factors; physical activity; smoking; alcohol; medication; and BMI. There were interactions between gender and B6 intake on EPA (*P-_interaction_* = 0.008) and AA (*P-_interaction_* = 0.004) only, whereas no interaction existed between gender and PLP on PUFA. PLP was directly associated with EPA (β = 0.181, *P* = 0.002), DHA (β = 0.109, *P* = 0.005), EPA + DHA (β = 0.14, *P* = 0.002), EPA/AA (β = 0.186, *P* = 0.004), and (EPA + DHA)/AA (β = 0.13, *P* = 0.026). The odds of having high plasma EPA (adjusted (a) OR: 2.03, *P* = 0.049) and EPA/AA (aOR: 3.83, *P* < 0.0001) were greater in those with adequate B6 status compared to those with deficient B6 status. In conclusion, in US older adults, a higher PLP level was associated with a greater level of EPA, DHA, EPA + DHA, EPA/AA, and (EPA + DHA)/AA. Adequate B6 status was associated with high EPA and EPA/AA status. These findings suggest that sufficient vitamin B6 status may positively influence PUFA metabolism in older adults.

## 1. Introduction

Polyunsaturated fatty acids (PUFA) are important constituents of phospholipids in cell membranes [1,2]. The structure and composition of membrane lipids influence cellular functions [1,2], and polyunsaturated fatty acids mediate the inflammatory process by altering the membrane fluidity and the production pattern of lipid mediators [3]. In particular, n-3 PUFA, such as eicosapentaenoic acid (EPA; 20:5n-3) and docosahexaenoic acid (DHA; 22:6n-3), serving as precursors of anti-inflammatory metabolites [3], have been associated with healthy aging [4]. They may be beneficial in preventing or treating chronic diseases, such as cardiovascular diseases, linked to aging and inflammation [4,5].

The metabolic interrelationship between PUFA and vitamin B6 (B6) [6,7,8,9,10,11,12] has been suggested ever since early animal studies demonstrated the improving effect of unsaturated fatty acids on acrodynia-like dermatitis resulting from vitamin B6 deficiency [13,14,15,16,17]. Animal studies have shown that vitamin B6 deficiency decreased the contents of n-3 PUFA, EPA, DHA, and n-6 PUFA, arachidonic acid (AA; 20:4n-6) in plasma and tissue phospholipids, implying that B6 deficiency may impair the interconversions from α-linolenic acid (ALA; 18:3n-3) to EPA and DHA, and from linoleic acid (LA; 18:2n-6) to AA [8,9,10,11,12]. Furthermore, suboptimal vitamin B6 status decreased plasma levels of EPA, DHA, and AA but tended to elevate the plasma n-6/n-3 PUFA ratio in a clinical study with 23 healthy young adults (20–40 years) [7]. These data suggest a possible adverse impact of vitamin B6 deficiency on PUFA compositions. 

It is worth noting that aging may affect the metabolism of PUFA [18,19,20,21,22,23,24,25,26] and vitamin B6, as measured by plasma pyridoxal 5’-phosphate (PLP) [27,28,29]. The proportions of LA and AA in liver microsomal membranes were higher in 22-month-old male rats (old) than in 1-month-old counterparts (young), with no differences in ALA, EPA, and DHA [24]. Another study showed higher proportions of ALA, LA, and AA and lower proportions of DHA in liver microsomes of 6-, 10-, and 25-month-old male rats than in those of 1-month-old animals, with no difference in EPA [23]. Furthermore, a population-based study with 2793 adolescents and adults (≥15 years) reported the positive associations between age and proportions of EPA and DHA in serum phospholipids [22]. A clinical study revealed a higher percentage of EPA—but not DHA and AA—of plasma total lipids in 25 older adults (≥65 years) compared with 26 young adults (18–30 years) [18]. Meanwhile, epidemiological studies indicated age-related changes in vitamin B6 status [27,28,29]. Plasma PLP levels decreased at a rate of approximately 4 nmol/L per 10-year increase in age among those not taking B6 supplements in the Baltimore Longitudinal Study of Aging with 617 men (18–90 years) [27]. Similarly, in a cross-sectional study with British men and women (≥65 years), PLP concentrations decreased with advancing age [28].

Interestingly, Bordoni et al. [26] showed higher liver microsomal content of LA, but lower contents of AA and DHA, in 20-month-old male rats fed vitamin B6-deficient diets than in control aged animals, with no differences in ALA and EPA. These results imply a potential coupled impact of aging and vitamin B6 deficiency on PUFA compositions [26]. Thus, together with the aforementioned evidence on the metabolic link between vitamin B6 and PUFA [6,7,8,9,10,11,12] and the age-related alteration in PUFA profiles [18,19,20,21,22,23,24,25,26] and vitamin B6 status [27,28,29], the aging process might influence the metabolic interaction between PUFA and vitamin B6. Moreover, altered PUFA profiles during vitamin B6 deficiency [8,9,10,11,12] could be associated with various age-related health conditions [4,5]; however, little is known about the interconnection between vitamin B6 and PUFA metabolism and aging.

In light of the data suggesting the metabolic interrelationship between vitamin B6 and PUFA [6,7,8,9,10,11,12] and the potential effect of gender on PUFA profiles [22,30,31,32,33,34], our recent report revealed gender differences in the association between plasma PLP and plasma PUFA—but no gender-specific relationship for vitamin B6 intake—in US young and middle-aged adults (20–59 years), using the National Health and Nutrition Examination Survey (NHANES) 2003–2004 [35]. Building on our previous research scheme to assess gender differences in the association between them, this present study aimed to explore whether or not there was a gender-specific association of vitamin B6 intake and plasma PLP concentration, respectively, with plasma PUFA concentrations in US older adults (≥60 years) from the same NHANES 2003–2004. We further examined the relationship of adequate vitamin B6 status (plasma PLP ≥ 20 nmol/L) with high plasma PUFA status (PUFA levels above medians) relative to deficient vitamin B6 status (plasma PLP < 20 nmol/L) in older adults.

## 2. Materials and Methods

### 2.1. Data Source and Study Population

The National Center for Health Statistics (NCHS) conducts the NHANES to obtain nationally representative information on the health and nutritional status of the civilian non-institutionalized US population [36]. Written informed consent was obtained from all participants [37], and the survey protocol was approved by the NCHS Research Ethics Review Board [38].

Since the data on vitamin B6 intake, plasma PLP, and plasma PUFA together are uniquely available in the NHANES 2003–2004 cycle, this cross-sectional study utilized the 2003–2004 survey cycle that follows a stratified, multistage, clustered probability sampling design [36]. Trained personnel interviewed selected persons in their homes to collect demographic, socioeconomic, dietary supplement, and prescription drug data [37]. Then, trained staff collected anthropometric, dietary, reproductive history, and health-related data of the participants invited to the mobile examination center (MEC) [37]. Blood specimens were collected and processed in the MEC laboratory; laboratories under contract with NCHS conducted specimen testing for analysis [39]. 

**Figure 1:**
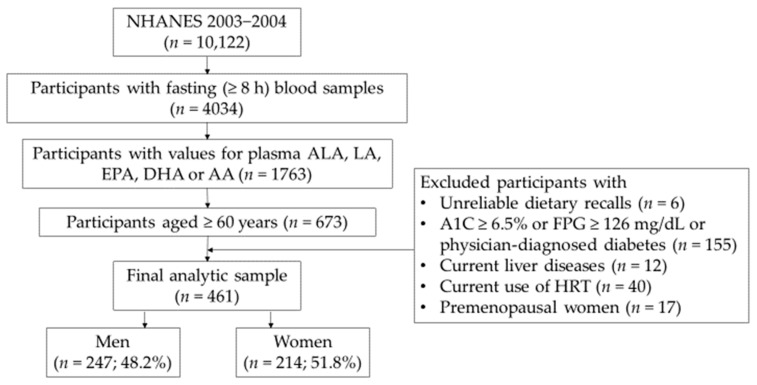
Flow chart of participant selection for the study population, NHANES 2003–2004. *n*, unweighted sample sizes; %, sample-weighted percentage; A1C, hemoglobin A1C; FPG, fasting plasma glucose; HRT, hormone replacement therapy; NHANES, National Health and Nutrition Examination Survey.

Figure 1 shows the sample selection process as a flow chart. A total of 10,122 participants completed the survey in the NHANES 2003–2004. 4034 participants aged ≥20 years who provided fasting blood samples (≥8 h) were included in this study. Of these, participants having measurements of plasma ALA, LA, EPA, DHA, or AA concentrations numbered 1763. Of these, 673 participants aged ≥60 years were included. Then, six participants with unreliable dietary recalls were excluded. Diabetes [40], current liver diseases [40], and hormone replacement therapy (HRT) [41] may alter plasma PLP levels. HRT may also influence plasma PUFA levels [42]. Thus, we excluded participants with physician-diagnosed diabetes or A1C ≥ 6.5% or fasting plasma glucose ≥126 mg/dL (*n* = 155), current liver diseases (*n* = 12), or current HRT use (*n* = 40). In addition, 17 premenopausal women were excluded since the small sample size was not adequate for inclusion in multivariate analyses. As a result, the final resulting analytic sample size was 461 participants, consisting of 247 men and 214 women. Depending on nonpositive weights, missing data, and/or model covariates, 384 to 454 participants were available for analyses in this study.

### 2.2. Assessment of Vitamin B6 and PUFA Intakes 

The NHANES utilizes a 24-h recall method for assessing dietary intakes; trained staff administered a dietary recall interview for collecting two days of dietary intake data, with the first day’s data being collected in-person at MEC and the second day’s data being collected by telephone 3–10 d after the in-person interview [43]. Using the US Department of Agriculture (USDA)’s Food and Nutrient Database for Dietary Studies 2.0 (FNDDS 2.0) 2003–2004, food codes were assigned to all foods and beverages collected. The FNDDS 2.0 processed the food data and analyzed nutrient values corresponding to foods and beverages based on USDA National Nutrient Database for Standard Reference, Release 18 (SR18) [44]. For food mixtures, nutrient values were estimated using the retention factor recipe calculation method; for commercial mixtures, they were calculated using label information [44]. In addition, the FNDDS accounts for nutrient losses from cooking when calculating the nutrient values of a given food by applying the set of retention factors for quantifying the amounts of nutrients retained after cooking [44].

The interviewer recorded data on the use of vitamins, minerals, and other dietary supplements—such as names, ingredients, amounts, and serving sizes [45]—and asked participants about duration, frequency, and daily amount of supplement use in the past 30 days [46]. NCHS obtained the supplements’ label information from manufacturers or retailers, Internet, company catalogs, and the Dietary Supplement Label Database containing entire label contents from supplements marketed in the US [46]. Using the NHANES Dietary Supplement Database, participants who used dietary supplements containing ingredients of vitamin B6, EPA, DHA, and ALA were identified.

We estimated the mean daily intakes of vitamin B6, ALA, LA, EPA, DHA, AA, total fat, and total energy from the averages of the first and second-day dietary recall data. Next, the average daily intakes of vitamin B6, EPA, DHA, and ALA from dietary supplements were calculated based on the number of days of using the supplements, the amount consumed daily, and the serving size unit from the supplement product label. Then, total daily intakes of those nutrients from food and supplements were calculated by summing up the mean daily intakes from dietary recall data and the average daily intakes from dietary supplement data. 

### 2.3. Measurements of Plasma PLP and Plasma PUFA

The NHANES documentation describes the detailed laboratory procedures for measuring the plasma concentrations of PLP [47] and PUFA [48].

Plasma PLP concentration (nmol/L) was measured using a homogeneous, nonradioactive, enzymatic assay (A/C Diagnostics, San Diego, CA, USA). The mean intra-assay coefficient of variation (CV) was 7.8% to 8.3%, and the mean inter-assay CV from 12.0% to 13.1% [47]. The detection limit of 10.09 nmol/L divided by the square root of 2 (7.1 nmol/L) superseded the assay values below the lower detection limit [47,49]. 

For fatty acid concentration measurements (µmol/L), fasting blood samples (≥8 h) were collected from subjects aged ≥20 years. Plasma fatty acids were measured by gas chromatography–mass spectrometry; the modified Lagerstedt et al. method was used for measuring plasma total fatty acid concentrations [48]. The mean intra-assay CV (SD) for analytes was 9% (10%), and the mean inter-assay CV (SD) for them was 8% (10%) [48]. Since participants with missing data on any fatty acids would affect the calculation of EPA + DHA, EPA/AA, and (EPA + DHA)/AA, they were not included in this study [50]. 

#### Updated Plasma Fatty Acid Data in NHANES 2003–2004

The reevaluation of the NHANES Biospecimen Program (2021–2012) processes addressed procedural errors in the biospecimen data files between 1999 and 2018 [48]. As a result, the 2003–2004 plasma fatty acid data file was updated in April 2022, which was modified to remove <5% of the original data records (1845 sample subset of NHANES 2003–2004 plasma samples); however, there were no modifications in data values [48].

This present study analyzed the updated data. Since our previous report published in January 2021 [35] used the original fatty acid data, the results were compared between the original and revised data; the main findings regarding the association between vitamin B6 and PUFA for younger adults in the previous study did not change. In addition, this study discussed the estimates from the previous report [35], such as the proportions of vitamin B6 deficiency in younger men and women, which had a <1% difference between the original and revised data.

### 2.4. Study Covariates 

Plasma PUFA levels may be affected by demographic and socioeconomic factors, cigarette smoking status, alcohol consumption, prescription medication use, and BMI [51,52,53]; thus, those variables were included as covariates. The consumption of fatty acids and total fat can affect PUFA composition [50,54,55], so these factors were also selected as covariates. In addition, since this present study and our previous report [35] examined the relationship between vitamin B6 and PUFA in two different age groups from the same NAHNES cycle, this study used the same covariates in the previous report for consistency.

Demographic variables included age (50–59, 60–69, 70–79, ≥80 years), gender (men, women), race/ethnicity (non-Hispanic White, non-Hispanic Black, Hispanic (Mexican American, Other Hispanic), and Others). Socioeconomic variables were poverty income ratio (PIR ≤ 1.3, >1.3; computed by dividing family income by poverty threshold) and educational attainment (high school graduation or less; some college or higher). Body mass index (BMI) was categorized as <18.5, 18.5–24.9, 25.0–29.9, and ≥30.0 kg/m^2^. 

Dietary variables included total fat intake; total intakes of vitamin B6, ALA, EPA, DHA from food and supplements; and dietary intakes of LA and AA from food. Other covariates include the following: cigarette smoking status (a never smoker who smoked <100 cigarettes in life; a former smoker who did not smoke at the time of interview; a current smoker who reported ongoing smoking), alcohol consumption (an abstainer who had <12 drinks of alcoholic beverage in life; a former drinker who had ≥12 drinks in life or any one year, but none in the past 12 months; a current drinker who had ≥12 drinks in life and drank ≥1 time in the past 12 months), physical activity level (<500, 500–1000, ≥1000 metabolic equivalent of task (MET) min/week; the MET score calculated from the frequency and duration of household/yard work, transportation, and leisure-time), and prescription medication use (a positive response to the question of taking any prescription medication in the past month).

### 2.5. Terminology for Gender and Sex

Gender is a term that refers to socially and culturally constructed characteristics of men and women based on the identification of a person’s gender or sex, whereas sex pertains to biological attributes differing between men and women according to the reproductive system [56,57]. In health-related research, the term ‘gender’ has been used interchangeably with the term ‘sex’ [57]. In NHANES, during the household screening interview, trained staff collect the data regarding the gender of a person by asking whether the person is male or female, which is coded accordingly [58]; then, during the MEC interview, the gender of a person is also confirmed by an interviewer [37]. The variable gender used in NHANES is a binary category based on whether the respondents identify themselves as male or female [58,59]. Thus, this study used the term ‘gender’ instead of ‘sex’.

### 2.6. Statistical Methods

The normality of the distributions of plasma PLP and plasma PUFA (EPA, DHA, AA, EPA + DHA, EPA/AA, (EPA + DHA)/AA) was evaluated using quantile-quantile plots and skewness values. Those plasma variables were highly skewed, except plasma AA having a near-normal distribution. After plasma PLP and plasma PUFA variables were natural log-transformed, those variables improved normality, approaching near-normal. Since no material differences were observed between the results using the log-transformed plasma AA and those using the original metric one, the log plasma AA was included in the analysis. Dietary intakes of vitamin B6, ALA, LA, EPA, DHA, and AA were energy-adjusted using the residual method [60]. 

Descriptive statistics of the covariates by gender were estimated. For categorical variables, frequencies and sample-weighted percentages with standard errors (SE) were obtained; for continuous variables, arithmetic (nutrient intakes) or geometric means (plasma PLP and PUFA) with SE were obtained. Rao–Scott chi-square tests for categorical variables and *t*-tests for continuous variables were used to compare the characteristics between men and women. 

#### 2.6.1. Linear Regression Analyses 

Positive correlations existed between vitamin B6 intake (from food and supplements) and plasma PLP in all subjects (correlation coefficient (*ρ*) = 0.39), men (*ρ* = 0.36), and women (*ρ* = 0.42) (Table S1). Vitamin B6 intake influences plasma PLP status [40,41,61]; therefore, the inclusion of vitamin B6 intake and plasma PLP together as independent variables in models may result in the unreliable estimation of regression coefficients in the fitted model owing to possible multicollinearity [62]. Thus, we reported the results of the following regression analyses by using two separate models for vitamin B6 intake and plasma PLP each. After checking residual plots, log-transformed plasma PUFA and PLP were used in linear regression analyses. 

Models were established by the sequential introduction of the covariates. Model 0 was unadjusted. Model 1 was adjusted for demographic variables (age, race/ethnicity), gender (not included for gender-stratified analyses), BMI, and dietary variables (total intakes of EPA, DHA, and ALA from food and supplements, dietary intakes of LA and AA from food, total fat intake). Model 2 was adjusted for all variables in Model 1, plus socioeconomic variables (PIR, educational attainment), physical activity level, cigarette smoking status, alcohol consumption, and prescription medication use. 

To evaluate whether the relationships of vitamin B6 intake and plasma PLP with plasma PUFA were modified by gender, the interaction terms of gender*B6 intake and gender*PLP were included separately in fully adjusted linear regression models. If there was a significant interaction, we further assessed the associations of B6 intake and plasma PLP with plasma PUFA using gender-stratified linear regression analyses. If not, we evaluated the associations in men and women combined. 

Unstandardized regression coefficients (b) with 95% confidence intervals (CI) were estimated, and a standardized coefficient (β) from linear regression was interpreted as a change in log plasma PUFA concentration or ratio in standard deviation (SD) for 1 SD of change in log plasma PLP concentration. A coefficient of determination, R^2^, was computed to measure the percentage of the total variability in plasma PUFA that each model explains, adjusted for the number of independent variables in Models 1 and 2. Variance inflation factor (VIF) >10 is considered indicative of multicollinearity; multicollinearity was not detected among independent variables in fully adjusted linear regression models for all subjects (VIF: 7.5 for total EPA intake, 7.9 for total DHA intake, 4.1 for dietary LA intake, 1.1–3.3 for the others) and in gender stratification models for men and women each (VIF: 8.1–8.4 for total EPA intake, 9.0–9.1 for total DHA intake, 3.9–4.9 for dietary LA intake, 1.1–4.0 for the others).

#### 2.6.2. Logistic Regression Analyses

Plasma PUFA concentrations and ratios (original metric values) were dichotomized as high plasma PUFA status (‘high’ defined as PUFA levels >medians) and low plasma PUFA status (‘low’ as ≤median). Vitamin B6 adequacy is defined as plasma PLP ≥ 20 nmol/L for establishing the current estimated average requirements (EAR) and recommended dietary allowances (RDA) of the vitamin [63]; thus, plasma PLP concentrations were categorized as adequate (PLP ≥ 20 nmol/L) and deficient (PLP < 20 nmol/L) vitamin B6 status. Logistic regression was employed to examine the relationship between adequate vitamin B6 status and high plasma PUFA in men and women combined, using deficient vitamin B6 status as a reference.

Odds ratios (OR) and 95% CI of high plasma PUFA for adequate vitamin B6 status relative to deficient B6 status were estimated. A median with interquartile range (IQR) was reported for each plasma PUFA. The same proposed models in the linear regression analyses were applied in the logistic regression analyses for consistency. A generalized coefficient of determination, *R*^2^ (likelihood-based pseudo *R*^2^), was measured for each model. There was no multicollinearity among covariates in fully adjusted logistic regression models (VIF: 7.5 for total EPA intake, 7.9 for total DHA intake, 4.1 for dietary LA intake, 1.1–3.2 for the others).

Statistical analyses were conducted with SAS 9.4 (SAS Institute Inc., Cary, NC, USA), using SAS survey procedures and applying appropriate sample weights to account for the complex survey design of NHANES. A two-sided *P* value <0.05 was considered to be statistically significant. 

## 3. Results

### 3.1. Demographic, Socioeconomic, and Other Characteristics of Participants by Gender in Older Adults

The participants’ characteristics by gender are presented in Table 1. The proportions of men and women were similar. Gender was distributed similarly for age groups, with almost half of the participants being aged 60–69 years and approximately one-third being aged 70–79 years. There were no differences in the proportions of race/ethnicity between men and women, with more than four-fifths being non-Hispanic White for both genders. More men tended to be overweight than women, with more women being obese (*P* = 0.05). Men were more likely to graduate high school or have a higher educational degree than women (*P* = 0.006). Compared to men, women were more likely to have a lower family income (*P* = 0.036) and were less likely to be physically active (*P* = 0.001). Compared to women, men were more likely to smoke cigarettes in the past, with similar proportions of current smokers for both genders (*P* < 0.0001), and to currently consume alcohol, with a greater proportion of lifetime abstainers for women (*P* = 0.001). On the other hand, there were no differences in the uses of vitamin B6 and n-3 PUFA supplements and prescription medication between men and women.

### 3.2. Distributions of Vitamin B6 and PUFA Intakes and Plasma PUFA and PLP Concentrations by Gender in Older Adults 

Table 2 shows the distributions of energy-adjusted dietary intakes from food and total intakes from food and supplements of vitamin B6 and PUFA, plasma PUFA concentrations and ratios, and plasma PLP concentration by gender in older adults. Distributions of original metric nutrient intakes are displayed in Table S2. We utilized the residual method [60] for estimating the energy-adjusted nutrient intakes to adjust absolute nutrient intakes for total energy by regressing nutrient intakes on total energy intake, providing the measures of nutrient intakes independent of total energy intake. As a result, in men, energy-adjusted mean values decreased from original metric ones, whereas, in women, energy-adjusted ones increased from original metric ones. 

The mean total energy intake was higher in men than in women after adjusting for demographic (age, race/ethnicity), socioeconomic factors (PIR, educational attainment), physical activity level, cigarette smoking status, alcohol consumption, prescription medication use, and BMI (*P* = 0.002). Compared to women, dietary vitamin B6 intake tended to be higher in men (*P* = 0.055), and dietary AA intake was greater in men (*P* = 0.008). However, there were no differences in dietary intakes of ALA, LA, EPA, DHA, and total fat between men and women. The mean total intakes of EPA and DHA were higher in men than in women (*P* = 0.033 for both). Differences between men and women did not exist in total intakes of vitamin B6 and ALA.

We observed a substantial increase from 1.8 mg/d of mean dietary vitamin B6 intake from food (1.8 mg/d for men, 1.7 mg/d for women) to 8.8 mg/d of mean total vitamin B6 intake from food and supplements (7.4 mg/d for men, 10.1 mg/d for women) in older adults. More than half of the older adults used vitamin B6 supplements, with no differences in the proportions of vitamin B6 supplement use between men and women. These indicate that some supplement users were taking high vitamin B6 dosages. Among vitamin B6 supplement users (*n* = 229), 10 participants (four men and six women) consumed more than 99.8 mg/d of vitamin B6 supplements (the 95th percentile), up to 214.5 mg/d (median (IQR), mg/d: 2.9 (2.1–5.5) for all users; 2.7 (2.2–5.1) for men; 2.9 (2.0–5.5) for women; data not shown), which could influence the large increase in mean total vitamin B6 intakes. The mean plasma PLP concentration was more than twice as high in vitamin B6 supplement users as in non-users (79.1 ± 5.1 nmol/L for users vs. 30.0 ± 2.5 nmol/L for non-users, *P* < 0.0001; data not shown).

The geometric mean plasma PLP concentration was lower in women than in men after adjusting for demographic and socioeconomic factors, total energy intake, vitamin B6 intake, physical activity level, cigarette smoking status, alcohol consumption, prescription medication use, and BMI (*P* = 0.031). However, the mean plasma PLP concentrations for both men (58.4 nmol/L) and women (46.0 nmol/L) were above 20 nmol/L, which is considered adequate vitamin B6 status. Furthermore, there were similar proportions of vitamin B6 deficiency between the two genders, with 14.3% of men and 16.2% of women.

There were higher geometric mean plasma concentrations of LA, EPA, DHA, AA, and EPA + DHA in women than in men (*P* = 0.006, *P* = 0.005, *P* = 0.013, *P* = 0.002, *P* = 0.01, respectively) after adjusting for demographic and socioeconomic factors; physical activity level; cigarette smoking status; alcohol consumption; prescription medication use; BMI; total fat intake; total intakes of EPA, DHA, and ALA; and dietary intakes of LA and AA. However, there were no differences in mean plasma ALA concentration and mean plasma EPA/AA and (EPA + DHA)/AA ratios between men and women.

### 3.3. Associations of Vitamin B6 Intake with Plasma EPA and AA Concentrations, Stratified by Gender, in Older Adults

We evaluated whether gender modifies the associations between vitamin B6 intake (from food and supplements) and plasma PUFA concentrations and ratios in older adults. There were significant interactions between gender and vitamin B6 intake on plasma EPA (*P-_interaction_* = 0.008) and AA (*P-_interaction_* = 0.004). On the other hand, the interactions on DHA (*P-_interaction_* = 0.19), EPA/AA (*P-_interaction_* = 0.11), and (EPA + DHA)/AA (*P-_interaction_* = 0.66) were not significant; the interaction on EPA + DHA did not reach statistical significance (*P*-*_interaction_* = 0.08). These results indicate that the relationship between vitamin B6 intake and plasma concentrations of EPA and AA each—but not DHA, EPA + DHA, EPA/AA, and (EPA + DHA)/AA—differs by gender among older adults.

Due to the significant interactions, we assessed the relationships of vitamin B6 intake with plasma EPA and AA concentrations using gender-stratified bivariate and multivariate linear regression analyses, presented in Table 3. In Model 2, fully adjusted for demographic and socioeconomic factors, dietary factors (total fat intake; total intakes of EPA, DHA, and ALA; dietary intakes of LA and AA), BMI, physical activity level, cigarette smoking status, alcohol consumption, and prescription medication use, there was a positive association between vitamin B6 intake and log plasma EPA concentration in women (b = 0.003, *P* = 0.015). In contrast, a negative association between them was observed in men (b = −0.003, *P* = 0.02). It is interpreted that vitamin B6 intake increases by 0.139 SD for 1 SD increase in log plasma EPA concentration in women, but decreases by 0.151 SD in men, in the full Model 2. 

Based on the regression coefficients, the estimated effects of vitamin B6 intake on plasma EPA concentration changed between the unadjusted Model 0 and the full Model 2. In women, the standardized coefficient (β) for plasma EPA shifted from 0.152 in Model 0 to 0.139 in Model 2; in men, from −0.051 to −0.151. These suggest that demographic factors, socioeconomic factors, dietary factors, physical activity level, cigarette smoking status, alcohol consumption, prescription medication use, and BMI may mediate the association between vitamin B6 intake and plasma EPA concentration in both genders.

According to the coefficient of determination, in women, 25% of the variance in plasma EPA is predicted by demographic factors, dietary factors, and BMI in the partial Model 1 (adjusted (a) *R*^2^ = 0.25). After further adjustment in the full Model 2, the additional 13% of the variance in plasma EPA is further explained by socioeconomic factors, physical activity level, cigarette smoking status, alcohol consumption, and prescription medication use (a*R*^2^ = 0.38). In men, 22% of the variance in plasma EPA is explained by demographic, socioeconomic, and dietary factors; BMI; physical activity level; cigarette smoking status; alcohol consumption; and prescription medication use in the full Model 2 (a*R*^2^ = 0.22). 

In men, there were no significant associations between vitamin B6 intake and plasma AA concentration in all three models. However, in women, the association between them was significant in the partial Model 1 (b = 0.001, *P* = 0.039) but became marginally significant in the full Model 2 (b = 0.001, *P* = 0.07). 

In addition, we further explored the associations between vitamin B6 intake and plasma DHA, EPA + DHA, EPA/AA, and (EPA + DHA)/AA in older men and women combined. No significant associations between them existed, fully adjusted for age, race/ethnicity, gender, socioeconomic, and dietary factors; physical activity level; cigarette smoking status; alcohol consumption; prescription medication use; and BMI (data not shown).

### 3.4. Associations of Plasma PLP Concentration with Plasma PUFA Concentrations and Ratios in Older Adults

We tested whether the relationship of plasma PLP with plasma PUFA was modified by gender. No significant interaction existed between gender and plasma PLP on plasma EPA (*P-_interaction_* = 0.39), DHA (*P-_interaction_* = 0.42), AA (*P*-*_interaction_* = 0.09), EPA + DHA (*P-_interaction_* = 0.4), EPA/AA (*P-_interaction_* = 0.89), and (EPA + DHA)/AA (*P-_interaction_* = 0.997) each, indicating that the association of plasma PLP with plasma EPA, DHA, AA, EPA + DHA, EPA/AA, and (EPA + DHA)/AA, respectively, does not differ by gender. Thus, among older men and women combined, the relationships between plasma PLP concentrations and plasma PUFA concentrations and ratios were examined using bivariate and multivariate linear regression analyses, displayed in Table 4. 

In all three models, plasma PLP concentration was directly associated with plasma concentrations of EPA, DHA, EPA + DHA, and ratios of EPA/AA and (EPA + DHA)/AA in older adults. In the full Model 2, there were positive associations of plasma PLP with plasma EPA (b = 0.104, *P* = 0.002), DHA (b = 0.045, *P* = 0.005), EPA + DHA (b = 0.06, *P* = 0.002), EPA/AA (b = 0.096, *P* = 0.004), and (EPA + DHA)/AA (b = 0.052, *P* = 0.026). It is interpreted that log plasma EPA, DHA, EPA + DHA, EPA/AA, (EPA + DHA)/AA concentrations increase by 0.181 SD, 0.109 SD, 0.14 SD, 0.186 SD, 0.13 SD, respectively, for one SD increase in log plasma PLP concentration in Model 2. The standardized coefficient (β) for plasma EPA dropped from 0.215 in Model 0 to 0.181 in Model 2; for DHA, from 0.147 to 0.109; for EPA + DHA, from 0.178 to 0.14; for EPA/AA, from 0.252 to 0.186; and for (EPA + DHA)/AA, from 0.209 to 0.13. These results imply that, in older adults, the association of plasma PLP with plasma EPA, DHA, EPA + DHA, EPA/AA, (EPA + DHA)/AA, respectively, may be modulated by demographic (age, race/ethnicity, gender), socioeconomic, and dietary factors; physical activity level; cigarette smoking status; alcohol consumption; prescription medication use; and BMI.

In the partial Model 1, 17%, 22%, 22%, 17%, and 19% of the variance for plasma EPA, DHA, EPA + DHA, EPA/AA, (EPA + DHA)/AA, respectively, is predicted by demographic and dietary factors and BMI (a*R*^2^ = 0.17, a*R*^2^ = 0.22, a*R*^2^ = 0.22, a*R*^2^ = 0.17, a*R*^2^ = 0.19 each). In the full Model 2, the additional 7%, 8%, 8%, 6%, and 6% of the variance for plasma EPA, DHA, EPA + DHA, EPA/AA, (EPA + DHA)/AA each is further explained by socioeconomic factors, physical activity level, cigarette smoking status, alcohol consumption, and prescription medication use (a*R*^2^ = 0.24, a*R*^2^ = 0.3, a*R*^2^ = 0.3, a*R*^2^ = 0.23, a*R*^2^ = 0.25, respectively).

In contrast, the association between plasma PLP concentration and plasma AA concentration was not significant in any of the models.

Additionally, we assessed whether the relationship of plasma PLP concentration with plasma PUFA concentration was modified by vitamin B6 supplement use in the fully adjusted linear regression model. There were no significant interactions between vitamin B6 supplement use and plasma PLP on plasma PUFA, except for EPA/AA (*P*-*_interaction_* = 0.049). These results indicate that, overall, the association of plasma PLP with plasma PUFA did not differ by B6 supplement use in the study participants (data not shown).

### 3.5. Odds Ratios of High Plasma PUFA Status for Adequate Vitamin B6 Status Versus Deficient Vitamin B6 Status in Older Adults

Table 5 shows the odds ratios (OR) with 95% confidence intervals (CI) of high plasma PUFA status (above median) for adequate vitamin B6 status (plasma PLP ≥ 20 nmol/L) relative to deficient vitamin B6 status (plasma PLP < 20 nmol/L) in older men and women combined, which were assessed by bivariate and multivariate logistic regression analyses. There were no interactions between gender and vitamin B6 status on plasma PUFA status in fully adjusted logistic regression models.

Adequate vitamin B6 status was associated with greater odds of high plasma status of EPA and EPA/AA, respectively, among older adults in all three models. In the full Model 2, the odds of having high plasma EPA and EPA/AA were 2.03 times and 3.83 times greater each in those with adequate vitamin B6 status as compared to those with deficient vitamin B6 status (adjusted (a) OR: 2.03, *P* = 0.049 for EPA; aOR: 3.83, *P* < 0.0001 for EPA/AA).

In the unadjusted Model 0, there was an association between adequate B6 status and higher odds of high plasma (EPA + DHA)/AA status (aOR: 2.14, *P* = 0.017). After the partial adjustment for demographic and dietary factors and BMI (Model 1), their association remained significant (aOR: 2.5, *P* = 0.004). However, after the additional adjustment of socioeconomic factors, physical activity level, cigarette smoking status, alcohol consumption, and prescription medication use (Model 2), those with adequate B6 status tended to have greater odds of having high plasma (EPA + DHA)/AA status than those with deficient B6 status (aOR: 2.11, *P* = 0.054). 

The association between adequate vitamin B6 status and high plasma EPA + DHA status was marginally significant in the unadjusted Model 0 (aOR: 1.51, *P* = 0.053). Their association became significant in the partial Model 1 (aOR: 1.62, *P* = 0.027); then lost the significance in the full Model 2. Conversely, there were no associations between adequate vitamin B6 status and high plasma AA status in all models.

In addition, we evaluated if the association of vitamin B6 status with plasma PUFA status differed by B6 supplement use in the fully adjusted logistic regression model. No significant interactions existed between B6 supplement use and B6 status on plasma PUFA status, indicating that the association of vitamin B6 status with plasma PUFA status did not vary by vitamin B6 supplement use among older adults in this study (data not shown).

## 4. Discussion 

This present study revealed that, among US adults aged ≥60 years, plasma PLP concentration was positively associated with plasma concentrations of EPA, DHA, EPA + DHA, and ratios of EPA/AA, (EPA + DHA)/AA, respectively, after adjusting for demographic, socioeconomic, and dietary factors; physical activity level; cigarette smoking status; alcohol consumption; prescription medication use; and BMI. Furthermore, adequate vitamin B6 status was associated with greater odds of high plasma EPA and EPA/AA status each relative to deficient B6 status. The relationship of plasma PLP level with plasma PUFA level did not differ by gender, while there were gender differences in the relationships of vitamin B6 intake with plasma EPA and AA levels. To the best of our knowledge, this study is the first to report the associations of vitamin B6 intake and status, measured by plasma PLP, each with plasma PUFA in the nationally representative sample of the US older adult population.

The mean plasma EPA, DHA, and AA concentrations were higher in women than in men among older adults in this study. This result may be partially, but not entirely, supported by studies demonstrating higher DHA—but lower EPA—proportions of serum phospholipids in women than in men between the ages of 55 years and 73 years [22]; and a higher percentage of plasma AA in women than in men, with no difference in DHA, among adults (≥51 years) [64]. In addition, epidemiological evidence indicated the age-related decrease in PLP concentration [27,28]. This study showed that the mean plasma PLP concentration for men (58.4 nmol/L) and women (46.0 nmol/L) were above the normal range, which might be possibly due to the result that more than half of the study participants were vitamin B6 supplement users (men: 57%, women: 55%). This could be, in part, explained by studies reporting that the age-associated reduction in circulating PLP levels was diminished by vitamin B6 supplementation [27,40,41].

The physiological pathways involving Δ6 desaturase (D6D) [7,65] may, to some extent, explain this study’s finding of the positive associations of plasma PLP concentration with plasma EPA, DHA, EPA + DHA concentrations and EPA/AA, (EPA + DHA)/AA ratios in older adults. PUFA synthesis is regulated by D6D [6,11,26,66], a rate-limiting enzyme converting LA to AA and ALA to EPA and DHA. Inadequate vitamin B6 status may inhibit longer chain PUFA production through the altered activity of D6D [6,11,26,66], influenced by the status of PLP [61,66,67]. Furthermore, animal [23,24] and human [20] studies have indicated that aging may affect the activity of D6D. Hrelia et al. [23] demonstrated the decreased liver microsomal D6D activity for LA and ALA in 25-month-old male rats (old) compared to 1-month-old animals (young) [24]. Product-to-substrate ratios (an indicator of D6D activity) of AA/LA and n-3 PUFA/ALA were lower in 6-, 10-, and 25-month-old rats than in 1-month-old animals [24]. Furthermore, the Scottish Heart Health Study with 2308 men and 2049 women (40–59 years) reported that LA/GLA (gamma-linolenic acid; 18:3n-6) had a significant positive association with age in men, with a non-significant positive association in women [20]. These data indicate that the declined D6D activity appeared to occur with advancing age [20,23,24]. Moreover, a possible adverse impact of aging and vitamin B6 deficiency on longer chain PUFA synthesis was suggested by the study showing that the D6D activity for LA and ALA in liver microsomes was lower in vitamin B6-deficient aged male rats (20-month-old) than in control aged animals [26]. The evidence so far may imply that vitamin B6 status might influence fatty acid desaturation and composition in an age-dependent manner, in part, by regulating the activity of D6D.

Nevertheless, the metabolic interplay between vitamin B6 and PUFA during aging has not been fully clarified. Data from animal and human studies on the relationship between aging, vitamin B6, and PUFA are sparse. Several epidemiological studies on vitamin B6 and PUFA used different types of study designs (i.e., clinical trial, prospective or observational study) and had various study target populations, such as healthy adults (20–40 years or 21–60 years) [6,7]; pregnant women [61]; children/adolescent or adolescent only [65,68]; and patients with suspected coronary heart disease (median age 61 y) [69] or with depressive disorders and healthy controls (18–65 years) [70]. Some studies showed positive associations between circulating PLP and n-3 PUFA, EPA, or DHA [61,69], while another study reported no association [70]. It appears that, due to the differences in study designs, participants, and age groups, those studies yielded mixed results on the relation between vitamin B6 status and blood PUFA. Despite the lack of directly comparable evidence involving older adults that could closely match this study’s participants, most studies described above suggest that vitamin B6 status may alter endogenous PUFA metabolism [6,7,61,65,69]. The evidence thus far might partially support this study’s finding of the positive relationship between plasma PLP and plasma PUFA in older adults. However, insufficient data exist to corroborate this study’s finding on the association between them; therefore, future research on the link between vitamin B6 and PUFA status in the older population is needed to confirm the results of this study.

There were no gender differences in the associations of plasma PLP with plasma EPA, DHA, EPA + DHA, EPA/AA, and (EPA + DHA)/AA among older adults (≥60 years) in this study. In contrast, we previously reported gender differences in the associations of plasma PLP with those PUFA among young and middle-aged adults (20–59 years), with significant direct associations in men only [35]. We speculated that the lower status of both vitamin B6 and iron in younger women than in men might contribute to the gender-specific relationship between plasma PLP and plasma PUFA in younger adults [35]. The deficiency or depletion of iron, present in the terminal protein of the D6D enzyme [71], may modify PUFA metabolism [72,73,74,75,76]. Furthermore, D6D activity may be impaired by vitamin B6 deficiency, thereby inhibiting the synthesis of longer chain PUFA [6,11,26,66]. This prior evidence suggests that vitamin B6 [6,11,26,66] and iron [72,73,74,75,76] may interact with PUFA metabolism. It is noteworthy that, in older adults, there were similar proportions of vitamin B6 deficiency (men: 14%, women: 16%) and low iron status (men: 16%, women: 17%; Table S3) between men and women. On the other hand, in younger adults, the proportions of both vitamin B6 deficiency and low iron status were higher in younger women than in men (B6 deficiency: 29% for women, 10% for men; low iron status: 28% for women, 14% for men) [35]. Thus, the nonexistence of gender-specific associations between plasma PLP and plasma PUFA for older adults observed in this study might be, in part, attributable to the similar prevalence of vitamin B6 deficiency and low iron status between older men and women. However, given the limited data on the metabolic connection between vitamin B6, iron, and PUFA with advancing age, it remains to be answered whether the age-associated changes in vitamin B6 and iron status could modify circulating PUFA compositions. 

In addition, unlike no gender differences in the relationship of vitamin B6 intake with plasma PUFA in younger adults reported in our previous study [35], this study showed the associations of vitamin B6 intake with plasma EPA and AA differed by gender in older adults. Perhaps these findings might be associated with this study’s results of no difference in the mean vitamin B6 intakes between men and women but the higher mean plasma EPA and AA levels in women compared with men. To a certain extent, they might reflect a possible influence of age on gender differences in PUFA profiles in older individuals [64], even with the lack of data on age- and gender-related changes in vitamin B6 intake and PUFA metabolism.

The associations of adequate vitamin B6 status with high plasma EPA and EPA/AA status relative to deficient B6 status in older adults were observed in this study. Age-related alterations in PUFA metabolism [18,19,20,21,22,23,24,25,26] may be linked with inflammation-associated diseases, particularly cardiovascular diseases (CVD) related to aging [3,77]. EPA and AA are involved in regulating the inflammatory process by serving as precursors to anti-inflammatory and pro-inflammatory/pro-aggregatory eicosanoids, respectively [78]. EPA reduces the generation of AA-derived eicosanoids with their inhibitory effects on AA metabolism [78]. The inverse relationships of circulating EPA with the markers of inflammation and the CVD incidence were shown in the Multi-Ethnic Study of Atherosclerosis [79]; plasma EPA and EPA/AA were inversely related to the risk of major coronary events in the Japan EPA Lipid Intervention Study [80]. The blood EPA/AA ratio has been regarded to be indicative of chronic inflammation and CVD risk [81,82]. Moreover, the inverse association between vitamin B6 and inflammation has been reported [83,84], and PUFA profiles were altered by vitamin B6 deficiency [6,7]. Based on the evidence above, this study’s findings on the associations of adequate vitamin B6 status with high plasma EPA and EPA/AA may imply potential metabolic interactions between vitamin B6, PUFA, and inflammation; further, these findings may suggest that maintaining sufficient B6 status could favorably impact plasma PUFA compositions in older adults. Still, understanding the interrelationship between vitamin B6 and PUFA metabolism and inflammation in the older population is not clear yet, so this study highlights the need to elucidate the relationship between them.

The present study’s results are subject to several limitations. First, the nature of the cross-sectional study does not allow translation of the results into the cause-and-effect relationship. Second, the observed association between vitamin B6 and PUFA could still be influenced by unmeasured suspected confounders, such as vegetarianism, which may lower n-3 PUFA concentrations [85], and components involved in PLP-dependent metabolic reactions such as one-carbon metabolism (i.e., folate, vitamin B12, homocysteine). Third, the data on PUFA intakes from the 2003–2004 survey cycle used in this study could not reflect the increasing trend of fish oil supplement uses among US adults [86,87], which may influence the plasma PUFA status of the contemporary population. Fourth, the percentage of participants with plasma PUFA measurements is 44% of those with fasting blood samples since plasma fatty acids measurement was a part of a surplus specimen project of the 2003–2004 cycle, which may cause a sample selection bias in the study results. Lastly, the utilization of circulating PUFA and vitamin B6 biomarkers was limited to plasma fatty acids and PLP each since data for other sources of PUFA and vitamin B6—such as erythrocytes—were not available in the survey cycle that this study used. Accordingly, this might hinder reflecting the full aspects of the relationship between vitamin B6 and PUFA. 

Despite these limitations, there are strengths of this study. First, using two-day non-consecutive 24-h dietary recalls (the second day dietary data collected 3 to 10 days after the first day 24-h recall) in this study allows for minimizing within-person variability of nutrient intakes [88] although 24-h recalls are associated with measurement error [89]. Second, the utilization of multiple confounding factors from the NHANES data in the analysis made potential bias sources minimized. Third, the distinctive 2003–2004 survey cycle containing the data on vitamin B6 intake, plasma PLP, and plasma PUFA enabled the assessment of the relationship between vitamin B6 intake and status and plasma PUFA in the US representative sample. 

In conclusion, among US older adults, the higher plasma PLP concentration was associated with the greater plasma concentrations of EPA, DHA, EPA + DHA, and ratios of EPA/AA, (EPA + DHA)/AA, respectively, with no gender difference in the relationship between plasma PLP and plasma PUFA. Furthermore, adequate vitamin B6 status was associated with high plasma EPA and EPA/AA status. These findings suggest that sufficient vitamin B6 status may positively influence PUFA metabolism, which may confer health benefits to the older population. Future investigations are warranted to confirm the findings of this study.

## Figures and Tables

**Table 1:** Demographic, socioeconomic, and other characteristics of participants by gender among US adults aged ≥60 years, NHANES 2003–2004.

Characteristics	All (*n* = 461)			Men (*n* = 247)			Women (*n* = 214)			*P* ^1^
	*n*	% ± SE		*n*	% ± SE		*n*	% ± SE		
Gender	461	100 ± 0		247	48.2 ± 2.7		214	51.8 ± 2.7		0.51
Age (years)										0.39
60–69	195	49.6 ± 3.5		100	50.7 ± 5.1		95	48.5 ± 3.1		
70–79	148	32.2 ± 2.1		91	33.2 ± 3.5		57	31.2 ± 2.1		
80+	118	18.2 ± 2.1		56	16.1 ± 2.2		62	20.3 ± 2.9		
Race/Ethnicity										0.65
Non-Hispanic White	292	85.2 ± 2.8		153	83.7 ± 3.0		139	86.7 ± 2.9		
Non-Hispanic Black	58	6.9 ± 1.8		34	7.7 ± 1.9		24	6.2 ± 2.0		
Hispanic	100	4.7 ± 1.9		53	5.0 ± 2.0		47	4.5 ± 2.0		
Others	11	3.2 ± 0.8		7	3.7 ± 0.7		4	2.7 ± 1.3		
BMI (kg/m^2^)										0.050
≤24.9	141	31.5 ± 4.3		74	28.3 ± 3.4		67	34.6 ± 5.8		
25–29.9	191	42.1 ± 3.9		114	47.9 ± 3.9		77	36.8 ± 4.6		
≥30	122	26.3 ± 2.1		57	23.8 ± 3.0		65	28.7 ± 3.1		
PIR										0.036
≤1.3	128	19.4 ± 2.4		61	14.7 ± 2.9		67	23.8 ± 3.3		
>1.3	307	80.6 ± 2.4		177	85.3 ± 2.9		130	76.2 ± 3.3		
Educational attainment										0.006
≤High school degree	280	51.0 ± 3.1		141	44.1 ± 2.9		139	57.5 ± 4.3		
>High school degree	179	49.0 ± 3.1		105	55.9 ± 2.9		74	42.5 ± 4.3		
Physical activity (MET min/week)										0.001
<500	223	42.5 ± 3.2		101	34.6 ± 3.5		122	49.9 ± 5.1		
500–1000	61	14.8 ± 1.3		29	11.9 ± 1.9		32	17.6 ± 1.9		
≥1000	177	42.6 ± 2.3		117	53.6 ± 3.0		60	32.5 ± 3.9		
Cigarette smoking										<0.0001
Never smoker	207	42.5 ± 4.9		81	31.0 ± 5.5		126	53.2 ± 5.2		
Former smoker	200	45.7 ± 3.7		131	56.7 ± 3.9		69	35.5 ± 4.5		
Current smoker	54	11.8 ± 1.9		35	12.3 ± 2.2		19	11.3 ± 2.3		
Alcohol consumption										0.001
Lifetime abstainer	72	15.7 ± 3.3		18	8.6 ± 3.0		54	22.1 ± 4.5		
Former drinker	156	34.2 ± 2.7		86	35.8 ± 3.4		70	32.8 ± 2.6		
Current drinker	224	50.1 ± 4.8		134	55.6 ± 4.9		90	45.1 ± 5.3		
Vitamin B6 supplement										0.67
No	232	44.1 ± 2.7		129	42.9 ± 4.6		103	45.3 ± 3.2		
Yes	229	55.9 ± 2.7		118	57.1 ± 4.6		111	54.7 ± 3.2		
n-3 PUFA supplement										0.5
No	442	94.8 ± 1.4		235	93.8 ± 1.3		207	95.7 ± 2.2		
Yes	19	5.2 ± 1.4		12	6.2 ± 1.3		7	4.3 ± 2.2		
Prescription medication										0.98
No	90	17.8 ± 3.6		53	17.8 ± 3.1		37	17.9 ± 5.1		
Yes	371	82.2 ± 3.6		194	82.2 ± 3.1		177	82.1 ± 5.1		

BMI, body mass index; MET, metabolic equivalent of task; PIR, poverty income ratio; PUFA, polyunsaturated fatty acids; *n*, frequencies; %, sample-weighted percentages; SE, standard errors. Sample sizes are presented as unweighted. ^1^ Rao–Scott chi-square tests for examining whether there are differences in proportions between men and women across categories of each characteristic. The categories of BMI <18.5 kg/m^2^ and 18.5 < BMI ≤ 24.9 kg/m^2^ are combined into a category of BMI ≤24.9 kg/m^2^ since the frequency of BMI <18.5 kg/m^2^ in women is 0, which is not adequate for the chi-square test. The supplement use of vitamin B6 and *n*-3 PUFA is defined as a positive response to the question of taking any supplements containing vitamin B6 and *n*-3 PUFA (EPA, DHA, and ALA), respectively, in the past month.

**Table 2:** Distributions of energy-adjusted vitamin B6 and PUFA intakes and plasma PUFA and PLP concentrations by gender among US adults aged ≥60 years, NHANES 2003–2004.

	All (*n* = 461)		Men (*n* = 247)		Women (*n* = 214)		*P* ^1^
	*n*	Mean ± SE	*n*	Mean ± SE	*n*	Mean ± SE	
Nutrient intake from food							
Total energy (kcal/d)	424	1838.2 ± 47.9	221	2048.8 ± 79.7	203	1658.9 ± 37.1	0.002
Dietary vitamin B6 (mg/d)	424	1.78 ± 0.03	221	1.82 ± 0.04	203	1.74 ± 0.05	0.055
Dietary ALA (g/d)	424	1.49 ± 0.05	221	1.31 ± 0.05	203	1.41 ± 0.05	0.43
Dietary LA (g/d)	424	14.03 ± 0.35	221	13.37 ± 0.61	203	14.06 ± 0.65	0.3
Dietary EPA (g/d)	424	0.05 ± 0.01	221	0.05 ± 0.01	203	0.05 ± 0.01	0.25
Dietary DHA (g/d)	424	0.10 ± 0.02	221	0.10 ± 0.02	203	0.10 ± 0.02	0.13
Dietary AA (g/d)	424	0.13 ± 0.005	221	0.13 ± 0.01	203	0.12 ± 0.01	0.008
Total fat (g/d)	424	72.50 ± 1.18	221	71.32 ± 1.48	203	73.51 ± 1.59	0.47
Nutrient intake from food and supplements							
Total vitamin B6 (mg/d)	424	8.82 ± 1.51	221	7.37 ± 1.37	203	10.06 ± 2.28	0.32
Total ALA (g/d)	424	1.50 ± 0.05	221	1.43 ± 0.07	203	1.56 ± 0.06	0.31
Total EPA (g/d)	424	0.06 ± 0.01	221	0.07 ± 0.02	203	0.06 ± 0.01	0.033
Total DHA (g/d)	424	0.11 ± 0.02	221	0.11 ± 0.02	203	0.10 ± 0.02	0.033
Plasma Variables							
ALA (μmol/L)	456	67.81 ± 2.39	245	64.82 ± 2.60	211	70.71 ± 3.11	0.63
LA (μmol/L)	456	3514.9 ± 56.7	245	3346.6 ± 54.0	211	3679.0 ± 75.6	0.006
EPA (μmol/L)	457	49.09 ± 2.87	246	45.54 ± 3.05	211	52.64 ± 3.02	0.005
DHA (μmol/L)	457	140.8 ± 5.49	246	133.1 ± 6.03	211	148.4 ± 5.90	0.013
AA (μmol/L)	457	815.1 ± 9.81	246	767.1 ± 13.47	211	862.6 ± 11.93	0.002
EPA + DHA (μmol/L)	457	192.5 ± 8.18	246	181.2 ± 8.84	211	203.5 ± 8.64	0.01
EPA/AA	457	0.060 ± 0.003	246	0.059 ± 0.004	211	0.061 ± 0.003	0.35
(EPA + DHA)/AA	457	0.236 ± 0.008	246	0.236 ± 0.010	211	0.236 ± 0.009	0.73
PLP (nmol/L)	458	51.62 ± 3.89	246	58.36 ± 4.89	212	46.02 ± 3.83	0.031
PLP category ^2,3^							0.47
<20 nmol/L	87	15.3 ± 2.24	44	14.3 ± 2.41	43	16.2 ± 2.78	
≥20 nmol/L	371	84.7 ± 2.24	202	85.7 ± 2.41	169	83.8 ± 2.78	

AA, arachidonic acid; ALA, α-linolenic acid; DHA, docosahexaenoic acid; EPA, eicosapentaenoic acid; LA, linoleic acid; PLP, pyridoxal 5′-phosphate; PUFA, polyunsaturated fatty acids; SE, standard errors. Sample sizes (*n*) are presented as unweighted. Values are expressed as arithmetic means (nutrient intakes) or geometric means (plasma PUFA and PLP) with SE for continuous variables. Log-transformed values of plasma PUFA and PLP are used for *t*-tests. Number of observations used for *t*-tests: *n* = 389 for nutrient intakes; *n* = 386–387 for plasma PUFA; *n* = 384 for PLP. ^1^ *t*-test for comparing the means of dependent variables between men and women. ^2^ Rao–Scott chi-square tests to examine whether there is a difference in proportion between men and women for the PLP category. ^3^ Sample-weighted percentage (%) ± SE. For nutrient intakes: adjusted for demographic variables (age, race/ethnicity), BMI, socioeconomic variables (PIR, educational attainment), physical activity level, cigarette smoking status, alcohol consumption, and prescription medication use. For plasma PUFA: adjusted for demographic variables, socioeconomic variables, BMI, total fat intake, total intakes of EPA, DHA, and ALA; dietary intakes of LA and AA; physical activity level; cigarette smoking status; alcohol consumption; and prescription medication use. For plasma PLP: adjusted for demographic variables, socioeconomic variables, BMI, total energy intake, vitamin B6 intake, physical activity level, cigarette smoking status, alcohol consumption, and prescription medication use.

**Table 3:** Linear regression models: Associations of vitamin B6 intake with plasma EPA and AA concentrations, stratified by gender, among US adults aged ≥60 years, NHANES 2003–2004.

	Men (*n* = 247)				Women (*n* = 214)					
	β	b (95% CI)	*R* ^2^	*P*	β	b (95% CI)		*R* ^2^	*P*	*P*-*_int_.*^1^
	**Plasma EPA** (µmol/L)									
Vitamin B6 Intake (mg/d)										0.008
Model 0	−0.051	−0.001 (−0.004, 0.002)	0.003	0.45	0.152	0.004 (0.001, 0.006)		0.02	0.022	
Model 1	−0.141	−0.003 (−0.006, 0.001)	0.12	0.11	0.150	0.003 (0.001, 0.006)		0.25	0.017	
Model 2	−0.151	−0.003 (−0.005, −0.001)	0.22	0.02	0.139	0.003 (0.001, 0.005)		0.38	0.015	
	**Plasma AA** (µmol/L)									
Vitamin B6 Intake (mg/d)										0.004
Model 0	−0.080	−0.001 (−0.002, 0.001)	0.01	0.36	0.129	0.001 (−0.0001, 0.003)		0.02	0.06	
Model 1	−0.068	−0.001 (−0.002, 0.0004)	0.15	0.21	0.103	0.001 (0.0001, 0.002)		0.04	0.039	
Model 2	−0.072	−0.001 (−0.002, 0.0004)	0.21	0.23	0.104	0.001 (−0.0001, 0.002)		0.09	0.07	

AA, arachidonic acid; EPA, eicosapentaenoic acid; b, unstandardized regression coefficient; β, standardized regression coefficient; *P-int*., *P-interaction*; *R*^2^, a coefficient of determination; 95% CI, 95% confidence interval. Sample sizes (*n*) are presented as unweighted. Plasma PUFA variables are log-transformed. Standardized coefficients (β) are to be interpreted as a change in log-transformed plasma PUFA concentrations in standard deviation (SD) for 1 SD of change in vitamin B6 intake. Unadjusted *R*^2^ for Model 0; adjusted *R*^2^ for Models 1 and 2. Model 0: unadjusted. Model 1: adjusted for demographic variables (age, race/ethnicity), BMI, and dietary variables (total fat intake; total intakes of EPA, DHA, and ALA; dietary intakes of LA and AA). Model 2: adjusted for all variables in Model 1 plus socioeconomic variables (PIR, educational attainment), physical activity level, cigarette smoking status, alcohol consumption, and prescription medication use. ^1^ *P*-value for the interaction term gender*vitamin B6 intake on plasma PUFA added in the full Model 2. Number of observations used in the analysis of each model: Model 0: *n* = 220 for men, *n* = 202 for women; Model 1: *n* = 218 for men, *n* = 197 for women; Model 2: *n* = 206 for men, *n* = 181 for women.

**Table 4:** Linear regression models: Associations of plasma PLP concentration with plasma PUFA concentrations and ratios among US adults aged ≥60 years, NHANES 2003–2004.

	All (*n* = 461)			
	β	b (95% CI)	*R* ^2^	*P*
	**Plasma EPA** (µmol/L)			
Plasma PLP (nmol/L)				
Model 0	0.215	0.124 (0.066, 0.183)	0.05	0.0004
Model 1	0.208	0.121 (0.050, 0.192)	0.17	0.002
Model 2	0.181	0.104 (0.045, 0.163)	0.24	0.002
	**Plasma DHA** (µmol/L)			
Plasma PLP (nmol/L)				
Model 0	0.147	0.062 (0.025, 0.099)	0.02	0.003
Model 1	0.147	0.062 (0.028, 0.096)	0.22	0.002
Model 2	0.109	0.045 (0.016, 0.074)	0.30	0.005
	**Plasma AA** (µmol/L)			
Plasma PLP (nmol/L)				
Model 0	−0.022	−0.006 (−0.043, 0.032)	0.001	0.748
Model 1	0.031	0.008 (−0.032, 0.048)	0.09	0.674
Model 2	0.030	0.008 (−0.038, 0.054)	0.11	0.722
	**Plasma EPA + DHA** (µmol/L)			
Plasma PLP (nmol/L)				
Model 0	0.178	0.077 (0.038, 0.117)	0.03	0.001
Model 1	0.175	0.076 (0.035, 0.118)	0.22	0.001
Model 2	0.140	0.060 (0.026, 0.094)	0.30	0.002
	**Plasma EPA/AA ratio**			
Plasma PLP (nmol/L)				
Model 0	0.252	0.130 (0.072, 0.188)	0.06	0.0002
Model 1	0.218	0.113 (0.047, 0.179)	0.17	0.002
Model 2	0.186	0.096 (0.036, 0.157)	0.23	0.004
	**Plasma (EPA + DHA)/AA ratio**			
Plasma PLP (nmol/L)				
Model 0	0.209	0.083 (0.037, 0.130)	0.04	0.002
Model 1	0.171	0.068 (0.023, 0.114)	0.19	0.006
Model 2	0.130	0.052 (0.007, 0.097)	0.25	0.026

AA, arachidonic acid; DHA, docosahexaenoic acid; EPA, eicosapentaenoic acid; PLP, pyridoxal 5′-phosphate; PUFA, polyunsaturated fatty acids; b, unstandardized regression coefficient; β, standardized regression coefficient; *R*^2^, a coefficient of determination; 95% CI, 95% confidence interval. Sample sizes (*n*) are presented as unweighted. The plasma PUFA and PLP variables were log-transformed. Standardized coefficients (β) are to be interpreted as a change in log-transformed plasma PUFA concentrations and ratios in standard deviation (SD) for 1 SD of change in log-transformed plasma PLP concentration. Unadjusted *R*^2^ for Model 0; adjusted *R*^2^ for Models 1 and 2. Model 0: unadjusted. Model 1: adjusted for demographic variables (age, race/ethnicity, gender), BMI, and dietary variables (total fat intake; total intakes of EPA, DHA, and ALA; dietary intakes of LA and AA). Model 2: adjusted for all variables in Model 1 plus socioeconomic variables (PIR, educational attainment), physical activity level, cigarette smoking status, alcohol consumption, and prescription medication use. Number of observations used in the analysis of each model: Model 0: *n* = 454; Model 1: *n* = 412; Model 2: *n* = 384.

**Table 5:** Logistic regression models: Odds ratios of high (above median) plasma PUFA status for adequate vitamin B6 status (plasma PLP ≥ 20 nmol/L) versus deficient vitamin B6 status (plasma PLP < 20 nmol/L) among US adults aged ≥60 years, NHANES 2003–2004.

	All (*n* = 461)		
(Ref: Plasma PLP < 20 nmol/L)	OR (95% CI)	*R* ^2^	*P*
	High Plasma EPA		
Plasma PLP ≥ 20 nmol/L	[median (IQR), µmol/L: 49.9 (32.0–66.6)]		
Model 0	2.47 (1.35, 4.52)	0.02	0.003
Model 1	2.70 (1.19, 6.11)	0.14	<0.0001
Model 2	2.03 (1.00, 4.10)	0.20	0.049
	**High Plasma DHA**		
Plasma PLP ≥ 20 nmol/L	[median (IQR), µmol/L: 134.6 (107.0–187.4)]		
Model 0	1.24 (0.81, 1.88)	0.002	0.32
Model 1	1.48 (0.94, 2.34)	0.16	0.09
Model 2	1.36 (0.87, 2.13)	0.23	0.18
	**High Plasma AA**		
Plasma PLP ≥ 20 nmol/L	[median (IQR), µmol/L: 846.7 (694.4–979.1)]		
Model 0	0.80 (0.47, 1.36)	0.002	0.42
Model 1	0.73 (0.35, 1.51)	0.09	0.39
Model 2	0.65 (0.29, 1.46)	0.10	0.29
	**High Plasma EPA + DHA**		
Plasma PLP ≥ 20 nmol/L	[median (IQR), µmol/L: 185.9 (142.6–254.1)]		
Model 0	1.51 (1.00, 2.29)	0.01	0.053
Model 1	1.62 (1.06, 2.49)	0.16	0.027
Model 2	1.53 (0.95, 2.47)	0.22	0.08
	**High Plasma EPA/AA**		
Plasma PLP ≥ 20 nmol/L	[median (IQR): 0.059 (0.043–0.078)]		
Model 0	4.00 (2.32, 6.89)	0.05	<0.0001
Model 1	4.40 (2.30, 8.41)	0.12	<0.0001
Model 2	3.83 (1.97, 7.43)	0.16	<0.0001
	**High Plasma (EPA + DHA)/AA**		
Plasma PLP ≥ 20 nmol/L	[median (IQR): 0.215 (0.180–0.292)]		
Model 0	2.14 (1.15, 3.98)	0.02	0.017
Model 1	2.50 (1.34, 4.65)	0.13	0.004
Model 2	2.11 (0.99, 4.52)	0.18	0.054

AA, arachidonic acid; DHA, docosahexaenoic acid; EPA, eicosapentaenoic acid; PLP, pyridoxal 5′-phosphate; PUFA, polyunsaturated fatty acids; IQR, interquartile range; OR, odds ratio; Ref, reference; *R*^2^, likelihood-based pseudo *R*^2^; 95% CI, 95% confidence interval. Sample sizes (*n*) are presented as unweighted. The categorical variables of plasma PUFA and PLP are created using original metric values. Model 0: unadjusted. Model 1: adjusted for demographic variables (age, race/ethnicity, gender), BMI, and dietary variables (total fat intake; total intakes of EPA, DHA, and ALA; dietary intakes of LA and AA). Model 2: adjusted for all variables in Model 1 plus socioeconomic variables (PIR, educational attainment), physical activity level, cigarette smoking status, alcohol consumption, and prescription medication use. Number of observations used in the analysis of each model: Model 0: *n* = 454; Model 1: *n* = 412; Model 2: *n* = 384.

## Data Availability

The NHANES data used for these secondary analyses are publicly available at the webpage of the Centers for Disease Control and Prevention (CDC): https://wwwn.cdc.gov/nchs/nhanes/continuousnhanes/default.aspx?BeginYear=2003 (accessed on 29 April 2022).

## Supplementary Materials

The following supporting information can be downloaded at: https://www.mdpi.com/article/10.3390/nu14112336/s1, Table S1: Pearson correlation coefficients (*ρ*) between vitamin B6 intake and plasma PLP concentration by gender among US adults aged ≥60 years, NHANES 2003–2004; Table S2: Distributions of original metric intakes of vitamin B6 and PUFA by gender among US adults aged ≥60 years, NHANES 2003–2004; Table S3: Distributions of iron intake and serum iron levels by gender among US adults aged ≥60 years, NHANES 2003–2004.
