# Supplementary material for: Associations of Vitamin B6 Intake and Plasma Pyridoxal 5′-Phosphate with Plasma Polyunsaturated Fatty Acids in US Older Adults: Findings from NHANES 2003–2004
Source: Nutrients. 2022 Jun 2;14(11):2336. doi: 10.3390/nu14112336 (PMC9182930; doi:10.3390/nu14112336)
Supplement: Supplementary file 1 [file nutrients-14-02336-s001.zip › nutrients-1741529-supplementary.pdf]

## Supplementary Materials

**Table S1.** Pearson correlation coefficients ($\rho$) between vitamin B6 intake and plasma PLP concentration by gender among US adults aged ≥60 years, NHANES 2003–2004.

| | | **Vitamin B6 intake** (mg/d) |
|---|---|---|
| | | **ALL (*n*=461)** |
| **Plasma PLP** (nmol/L) | ***ρ* (rho)** | 0.39 |
| | ***P*** [1] | <0.0001 |
| | | **Men (*n*=247)** |
| **Plasma PLP** (nmol/L) | ***ρ* (rho)** | 0.36 |
| | ***P*** [1] | <0.0001 |
| | | **Women (*n*=214)** |
| **Plasma PLP** (nmol/L) | ***ρ* (rho)** | 0.42 |
| | ***P*** [1] | <0.0001 |

- PLP, pyridoxal 5′-phosphate.
- Sample size (*n*) is presented as unweighted.
- Log-transformed values of plasma PLP are used for Pearson correlation tests.
- [1] *P*-value for Pearson correlation tests.
- Number of observations used in the analysis: *n*=421 for all; *n*=220 for men; *n*=201 for women.

**Table S2.** Distributions of original metric intakes of vitamin B6 and PUFA by gender among US adults aged ≥60 years, NHANES 2003−2004.

| | All (*n*=461) | | Men (*n*=247) | | | Women (*n*=214) | | | |
|---|---|---|---|---|---|---|---|---|---|
| | ***n*** | **Mean ± SE** | ***n*** | **Mean ± SE** | **%RDA or %AI [3]** | ***n*** | **Mean ± SE** | **%RDA or %AI [3]** | ***P* [1]** |
| Nutrient intake from food | | | | | | | | | |
| Dietary vitamin B6 (mg/d) | 424 | 1.78 ± 0.04 | 221 | 1.95 ± 0.07 | (115%)[4] | 203 | 1.63 ± 0.05 | (109%)[4] | 0.012 |
| Dietary ALA (g/d) | 424 | 1.49 ± 0.06 | 221 | 1.63 ± 0.09 | (102%)[5] | 203 | 1.37 ± 0.08 | (125%)[5] | 0.52 |
| Dietary LA (g/d) | 424 | 14.0 ± 0.57 | 221 | 15.4 ± 0.75 | (n/a)[5] | 203 | 12.9 ± 0.65 | (n/a)[5] | 0.27 |
| Dietary EPA (g/d) | 424 | 0.05 ± 0.01 | 221 | 0.06 ± 0.01 | (n/a)[5] | 203 | 0.05 ± 0.01 | (n/a)[5] | 0.33 |
| Dietary DHA (g/d) | 424 | 0.10 ± 0.02 | 221 | 0.11 ± 0.02 | (n/a)[5] | 203 | 0.09 ± 0.02 | (n/a)[5] | 0.20 |
| Dietary AA (g/d) | 424 | 0.13 ± 0.01 | 221 | 0.14 ± 0.01 | (n/a)[5] | 203 | 0.11 ± 0.01 | (n/a)[5] | 0.005 |
| Total fat (g/d) | 424 | 72.50 ± 2.60 | 221 | 80.41 ± 4.30 | (n/a)[5] | 203 | 65.77 ± 2.30 | (n/a)[5] | 0.48 |
| Nutrient intake from food and supplements | | | | | | | | | |
| Total vitamin B6 (mg/d) | 424 | 8.82 ± 1.51 | 221 | 7.39 ± 1.38 | (435%)[4] | 203 | 10.04 ± 2.28 | (669%)[4] | 0.27 |
| Total ALA (g/d) | 424 | 1.50 ± 0.06 | 221 | 1.64 ± 0.09 | (103%)[5] | 203 | 1.39 ± 0.08 | (126%)[5] | 0.36 |
| Total EPA (g/d) | 424 | 0.06 ± 0.01 | 221 | 0.08 ± 0.02 | (n/a)[5] | 203 | 0.05 ± 0.01 | (n/a)[5] | 0.03 |
| Total DHA (g/d) | 424 | 0.11 ± 0.02 | 221 | 0.12 ± 0.02 | (n/a)[5] | 203 | 0.09 ± 0.02 | (n/a)[5] | 0.053 |

- AA, arachidonic acid; ALA, $\alpha$-linolenic acid; DHA, docosahexaenoic acid; EPA, eicosapentaenoic acid; LA, linoleic acid; PLP, pyridoxal 5′-phosphate; PUFA, polyunsaturated fatty acids; SE, standard error; DRI, dietary reference intakes; n/a, not available.
- Sample sizes (*n*) are presented as unweighted.
- Values are expressed as arithmetic means ± SE.
- [1] *t*-tests for comparing the means of dependent variables between men and women.
- Number of observations used for t-tests: *n*=389 for nutrient intakes.
- Adjusted for demographic variables (age, race/ethnicity), BMI, socioeconomic variables (PIR, educational attainment), physical activity level, cigarette smoking status, alcohol consumption, prescription medication use, and total energy intake.
- [3] %RDA or %AI is calculated by dividing each nutrient intake value by the corresponding RDA or AI value, then multiplying the obtained value by 100.
- [4] Recommended Dietary Allowance (RDA) for vitamin B6: 1.7 mg/d for men ≥51 y; 1.5 mg/d for women ≥51 y (Reference: Food and Nutrition Board Institute of Medicine A report of the Standing Committee on the Scientific Evaluation of Dietary Reference Intakes and its Panel on Folate, Other B Vitamins, and Choline and Subcommittee on Upper Reference Levels of Nutrients. Dietary reference intakes for thiamin, riboflavin, niacin, vitamin B6, folate, vitamin B12, pantothenic acid, biotin, and choline; National Academies Press: Washington, DC, 1998).
- [5] Adequate Intake (AI) for ALA: 1.6 g/d for men ≥51 y; 1.1 g/d for women ≥51 y; no AIs or RDAs for total fat, EPA, and DHA established by the Institute of Medicine (Reference: Institute of Medicine, Food and Nutrition Board. Dietary reference intakes for energy, carbohydrate, fiber, fat, fatty acids, cholesterol, protein, and amino acids (macronutrients). Washington, DC: National Academy Pres, 2005).

**Table S3.** Distributions of iron intake and serum iron levels by gender among US adults aged ≥60 years, NHANES 2003−2004.

| | All (*n*=461) | | Men (*n*=247) | | Women (*n*=214) | | |
|---|---|---|---|---|---|---|---|
| | *n* | Mean ± SE | *n* | Mean ± SE | *n* | Mean ± SE | *P* [1] |
| Original metric intake | | | | | | | |
| Dietary Iron (mg/d) | 424 | 15.17 ± 0.59 | 221 | 16.74 ± 0.76 | 203 | 13.83 ± 0.72 | 0.054 |
| Total Iron (mg/d) | 424 | 20.54 ± 0.97 | 221 | 22.99 ± 2.13 | 203 | 18.45 ± 1.31 | 0.46 |
| Energy-adjusted intake | | | | | | | |
| Dietary Iron (mg/d) | 424 | 15.17 ± 0.54 | 221 | 15.42 ± 0.43 | 203 | 14.95 ± 0.74 | 0.09 |
| Total Iron (mg/d) | 424 | 20.54 ± 0.83 | 221 | 21.75 ± 1.77 | 203 | 19.50 ± 1.42 | 0.61 |
| Serum iron (μmol/L) | 453 | 15.00 ± 0.40 | 242 | 16.41 ± 0.37 | 211 | 13.81 ± 0.51 | 0.0003 |
| Serum iron category [2,3] | | | | | | | 0.91 |
| Low | 89 | 16.6 ± 2.01 | 46 | 16.4 ± 2.09 | 43 | 16.9 ± 3.44 | |
| High | 364 | 83.4 ± 2.01 | 196 | 83.6 ± 2.09 | 168 | 83.2 ± 3.44 | |

- Sample sizes (*n*) are presented as unweighted.
- Values are expressed as arithmetic means (nutrient intakes) or geometric means (blood variables) ± standard error (SE) for continuous variables and sample-weighted percentages (%) with SE for categorical variables.
- Log-transformed values of serum iron and hemoglobin are used for *t*-tests.
- [1] *t*-tests for comparing the means of dependent variables between men and women.
- [2] Rao-Scott chi-square tests to examine whether there are differences in proportions between men and women across categories of each characteristic.
- [3] % ± SE.
- Sample sizes for t-tests: *n*=389 for iron intake; *n*=383 for serum iron; *n*=389 for hemoglobin.
- For iron intake: adjusted for demographic variables (age, race/ethnicity), BMI, socioeconomic variables (PIR, educational attainment), physical activity level, cigarette smoking status, alcohol consumption, prescription medication use, total energy intake (only for original metric dietary variables)
- For serum iron: adjusted for demographic variables (age, race/ethnicity), BMI, total iron intake, total energy intake, socioeconomic variables (PIR, educational attainment), physical activity level, cigarette smoking status, alcohol consumption, and prescription medication use.
- Low serum iron status is defined as <12 μmol/L for men and <10 μmol/L for women (Reference: Krajcovicova-Kudlackova M, Klvanova J, Dusinska M. Polyunsaturated fatty acid plasma content in groups of general population with low vitamin B6 or low iron serum levels. *Ann Nutr Metab*. 2004;48(2):118-121).
